# Synthesis of Xanthan Gum Anchored α-Fe_2_O_3_ Bionanocomposite Material for Remediation of Pb (II) Contaminated Aquatic System

**DOI:** 10.3390/polym15051134

**Published:** 2023-02-24

**Authors:** Fahad A. Alharthi, Riyadh H. Alshammari, Imran Hasan

**Affiliations:** Department of Chemistry, College of Science, King Saud University, Riyadh 11451, Saudi Arabia

**Keywords:** nanocomposite, heavy metal ion removal, Langmuir, chemical coprecipitation, wastewater treatment, adsorption

## Abstract

Increases in community and industrial activities have led to disturbances of the environmental balance and the contamination of water systems through the introduction of organic and inorganic pollutants. Among the various inorganic pollutants, Pb (II) is one of the heavy metals possessing non-biodegradable and the most toxic characteristics towards human health and the environment. The present study is focussed on the synthesis of efficient and eco-friendly adsorbent material that can remove Pb (II) from wastewater. A green functional nanocomposite material based on the immobilization of α-Fe_2_O_3_ nanoparticles with xanthan gum (XG) biopolymer has been synthesized in this study to be applied as an adsorbent (XGFO) for sequestration of Pb (II). Spectroscopic techniques such as scanning electron microscopy with energy dispersive X-ray (SEM-EDX), Fourier transform infrared (FTIR), transmission electron microscopy (TEM), X-ray diffraction (XRD), ultraviolet visible (UV-Vis) and X-ray photoelectron spectroscopy (XPS) were adopted for characterizing the solid powder material. The synthesized material was found to be rich in key functional groups such as –COOH and –OH playing important roles in binding the adsorbate particles through ligand-to-metal charge transfer (LMCT). Based on the preliminary results, adsorption experiments were conducted, and the data obtained were applied to four different adsorption isotherm models, viz the Langmuir, Temkin, Freundlich and D–R models. Based on the high values of R^2^ and low values of χ^2^, the Langmuir isotherm model was found to be the best model for simulation of data for Pb (II) adsorption by XGFO. The value of maximum monolayer adsorption capacity (Q_m_) was found to be 117.45 mg g^−1^ at 303 K, 126.23 mg g^−1^ at 313 K, 145.12 mg g^−1^ at 323 K and 191.27 mg g^−1^ at 323 K. The kinetics of the adsorption process of Pb (II) by XGFO was best defined by the pseudo-second-order model. The thermodynamic aspect of the reaction suggested that the reaction is endothermic and spontaneous. The outcomes proved that XGFO can be utilized as an efficient adsorbent material for the treatment of contaminated wastewater.

## 1. Introduction

The development of industrial production and population growth have had a huge impact on the contamination of surface and groundwater by toxic heavy metals and other pollutants [1,2]. These heavy metals are highly toxic and hazardous to the environment as well as mankind and can get taken up into the food chain [2,3,4]. Lead has been found to be a major hazardous heavy metal which is mainly generated by industrial activities such as mining, smelting, printing, and the production of dyes, rubber, lead glass, ceramics, solder, lead pipes and cables [4,5,6]. Due to high toxicity, excess Pb (II) can cause mutagenicity, carcinogenicity and teratogenicity, and Pb (II) contents are severely restricted in the environment by strict guidelines [7,8]. The United States Environmental Protection Agency (USEPA) has specified that the lowest contaminant level of Pb (II) is 0.015 mg L^−1^ [9,10]. Therefore, there is a huge concern for establishing an efficient technology for decontaminating heavy metals for the safety of mankind and the environment. Many techniques have been adopted, including ion exchange [11], adsorption [12], chemical oxidation [13], membrane separation [14] and precipitation [15], for decontaminating heavy metals. Among these methods, adsorption has been recognized as the most adequate and simplest method due to its ease of operation and cost-effective properties [16]. Developing an ideal adsorbent to fulfill the present demands is entirely related to its high efficiency, eco-friendly nature, simple reconstruction and separation [17].

Natural materials are completely harmless to the environment but fail to meet the requirements of perfect adsorption materials, and modification in parameters, such as abundant adsorption sites, material durability and recovery efficiency, are desirable [18,19,20]. Thus, the improvement of adsorbents to enhance their adsorption efficiency for lead ions has been the main center of attraction for many researchers in recent years. Therefore, nanomaterial-based adsorbent materials were considered due to their high aspect ratio and high efficiency to remove metal ions from wastewater [20]. So far, various adsorbent materials, such as carbon nanotubes [21], biochar [22], metal oxide nanoparticles [23] and metal organic frameworks (MOFs) [24], have been prepared for the sequestration of Pb (II) from wastewater. Among these, metal oxide nanoparticles have been explored and debated as the most efficient adsorbents due to their high surface area and small particle size. Among these, hematite nanoparticles (α-Fe_2_O_3_) have been found to be the most efficient in a broad range of applications such as biomedical science, energy production/storage, environment remediation and catalysis [25]. α-Fe_2_O_3_ NPs possess effective adsorption area at the nanometer scale with a size hundreds of times greater than α-Fe_2_O_3_ at the micron scale, and due to this property, they show very sensitive reactions at very low magnetic fields [26]. The nanostructured α-Fe_2_O_3_ has been reported to be an excellent regulator and adsorbent for the removal of various heavy metal ions such as Cr (VI), Co (II), Pb (II) and As (V), due to their less toxic and environmental friendly nature [27]. However, aggregation of nanoparticles leads to hindering of the adsorption efficiency of the material due to reduction in mobility, availability and transport to the adsorption site for the contaminated site [28]. Therefore, immobilization of nanoparticles by a multifunctional biopolymer was taken into consideration, which not only increased the biocompatibility of the synthesized material but also increased the adsorption capacity regarding extraction of heavy metal ions from wastewater.

Hybridizing natural polymers with nanofillers originated a new class of materials with better process-ability, biodegradability and effectiveness [29,30]. Naturally derived carbohydrate-based polymers possess remarkable properties of bio-compatibility, biodegradability, non-toxicity, hydrophilicity, worthwhile and recyclable nature and renewability, due to which they have become the main centre of attention globally [29,31]. Xanthan gum (XG) has been preferred in a high magnitude for adsorption of organic dyes and heavy metals due to the presence of glucuronic acid and pyruvic acid groups composed of abundant hydroxyl groups [32,33,34]. The presence of generous hydroxyl groups makes this biopolymer non-toxic, but its use is limited due to the water-solubility factor and low thermal and mechanical stability. These issues can be addressed through reinforcement by inorganic fillers (nanoparticles), which enhances their physical and chemical stability [35].

The present study explores the adsorption characteristics of XG-encapsulated α-Fe_2_O_3_ NPs towards Pb (II) with adsorption mechanism and the regeneration. Characterization of the synthesized material was performed using various spectroscopic techniques. The study also observed the effect of operational parameters such as pH and adsorbent dosage.

## 2. Materials and Methods

### 2.1. Synthesis of XG Capsuling α-Fe_2_O_3_ (XGFO) BNC

**Scheme 1:**
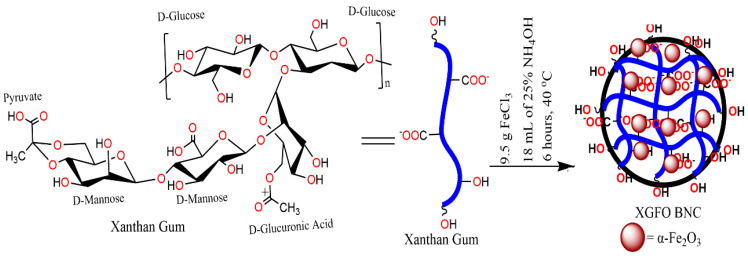
General Scheme for synthesis of the XGFO.

The chemicals used in this study were of analytical grades and used without any further purification. The BNC materials were synthesized by ecological green routes applying the chemical coprecipitation scheme [36]. A 100 mL solution of 9.5 g FeCl_3_.6H_2_O (Merck) was taken in a three-necked round bottom flask under continuous stirring for about 30 min on a magnetic stirrer (900 rpm) to obtain homogeneity. Another solution was made by taking 1.5 g of XG in 60 mL of distilled water dissolved at 40 ℃. The homogenous solution of XG was added slowly to the above-prepared FeCl_3_.6H_2_O solution with 18 mL of 25% ammonia drop by drop to maintain the pH of the system around 9–10. The system was left on magnetic stirring for 6 hrs and a dark brown precipitate was collected using a centrifuge (REMI rpm 8500). Removal of nonreactive species was made by squeezing the product with distilled water five to six times and further dried in an oven for 4 h at 50 °C. Scheme 1 represents the general scheme for the synthesis of the nanomaterial.

### 2.2. Analytical Techniques Used for Nanoparticle Characterization

Various characterization techniques are applied for nanoparticle synthesis and identification of crystal structure. The functional groups and type of bonding present in the synthesized product were observed by a Perkin Elmer spectrum 2 FTIR spectrometer. The surface morphology and shape of the nanocomposite material was observed by scanning electron microscopy (SEM, JEOL GSM 6510LV) and transmission electron microscopy (TEM, JEM 2100). The information about crystal phase and lattice structure and miller indices was evaluated by the X-ray diffraction method using a Rigaku Ultima 1V XRD spectrometer. The absorbance measurement was done through UV-1900 Shimadzu spectrophotometer. A Shimadzu UV-1900 UV-Vis double-beam spectrophotometer was used for taking the spectra of nanoparticles. The remaining concentration of Pb (II) after completion of the adsorption reaction was observed by atomic absorption spectrometer (AAS) GBC scientific instruments.

### 2.3. Adsorption Experiments and Design

Batch experiments were designed to carry out the adsorption experiments in an aqueous solution at a pH range of 3–5 and temperature of 303–333 K with an adsorbent dose (10–20 mg) and Pb (II) concentration of 120–150 mg L^−1^. The adsorbent was separated out of solution using centrifugation, and the supernatant collected was tested by AAS to assess the remaining concentration of PB (II). The data obtained after adsorption experiments were applied to various isotherms and a kinetic model for simulation and validation statistically. The efficiency of the synthesized material XGFO was calculated in terms of adsorption capacity, which is given by Equation (1):

(1) $$q_{e}=\frac{(C_{0}-C_{e})V}{W}$$

Here, *q_e_* represents the efficiency of the synthesized adsorbent material (mg g^−1^); *C*_0_ and *C_e_* represent concentrations of the metal ion in the initial solution (mg L^−1^) and after adsorption, respectively; *V* represents volume of the adsorption medium (L); *W* represents amount of the adsorbent (g).

### 2.4. Statistical Verification of Data

To obtain results with good precision, a statistical error analysis tool is combined with obtained data in order to get a decisive model that plays a demanding role in the photo-degradation reaction. Therefore, an association of regression coefficient (R^2^) with root mean square error (RMSE) was considered for the validation of experimental data for the most suitable model. Mathematically, RMSE can be given by Equation (2) [37]:

(2) $$\mathrm{RMSE}=\sqrt{\sum_{i=1}^{n}(q_{e,}{}_{\mathrm{cal}}-q_{e,\exp})i^{2}}$$

## 3. Results and Discussion

### 3.1. Adsorbent Characterization

**Figure 1:**
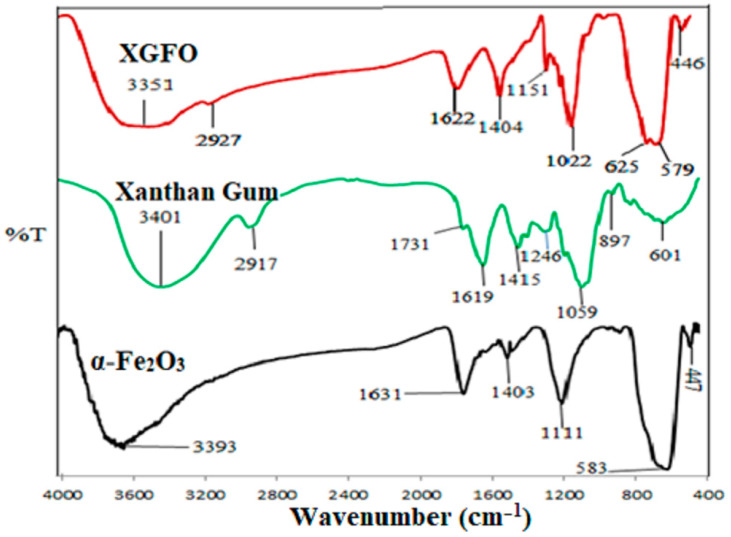
FTIR spectra of α-Fe_2_O_3_ (black line), Xanthan Gum (green line), XGFO (red line).

Figure 1 exhibits the FTIR spectra of α-Fe_2_O_3_, XG and XGFO BNC. The FTIR spectra band of α-Fe_2_O_3_ NPs represents characteristic peaks at 583 cm^−1^ (Fe–O stretching vibrations), 3393 cm^−1^, and 1631 cm^−1^ (O–H stretching and bending vibrations) [38]. The corresponding peaks of XG in FTIR spectra are found at 3401 cm^−1^ (O–H stretching), 2917 cm^−1^ (aliphatic –CH_2_ stretching), 1731 cm^−1^ (C=O stretching vibrations), 1619 and 1415 cm^−1^ (symmetric and asymmetric stretching COO^−^) [39]. The FTIR spectra of XGFO represent maxima of peaks from both XG and α-Fe_2_O_3_ with some shift in values of vibrational frequencies such as 446–625 cm^−1^ (Fe–O bond stretching); 1022–1151 cm^−1^ (C–O–C XG pyranoid ring); 1404 cm^−1^ (COO^−^ asymmetric stretching and asymmetric stretching) and 1622 cm^−1^ for C=O stretching. Shifts in the vibrational frequency of –COO and –OH groups suggested the involvement of these groups in stabilization of α-Fe_2_O_3_ NPs through Fe (III)–O–H type bond formations [40,41].

**Figure 2:**
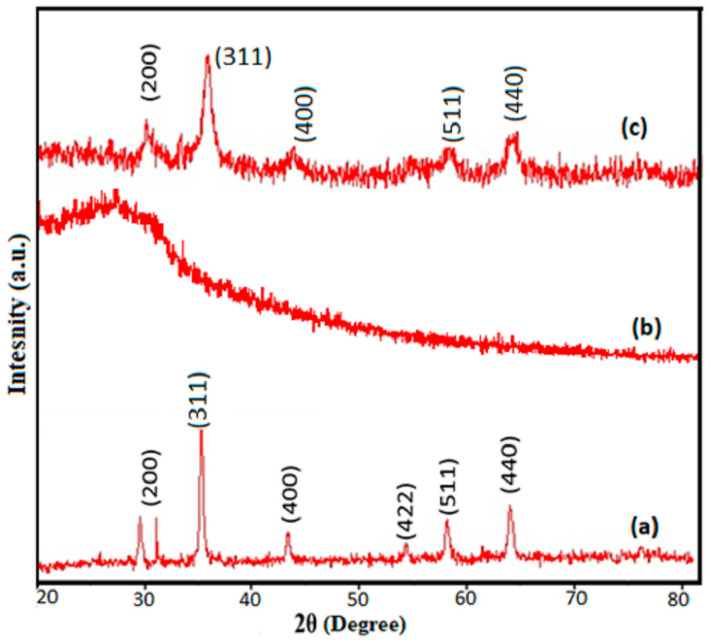
XRD Spectra of (**a**) α-Fe_2_O_3_ (**b**) XG and (**c**) XGFO.

The XRD spectra of pristine α-Fe_2_O_3_, XG and XGFO are given in Figure 2. The characteristic XRD peaks of pristine α-Fe_2_O_3_ NPs (Figure 2a) appeared at 2θ values of 31.89, 35.21, 44.27, 54.76, 57.47, and 63.05 simulated with Miller indices data with JCPDS No. 89-0510 having a rhombohedral crystalline structure. Figure 2b represents the XRD spectra of XG which shows a completely amorphous character with a peak around 26.67°. The XRD spectra of XGFO given by Figure 2c exhibited the pattern of α-Fe_2_O_3_ with reduced intensities of peaks and a semicrystalline character, suggesting the immobilization of α-Fe_2_O_3_ NPs by XG biopolymer chains. The reduction in peak intensities and appearance of amorphous character in the XRD spectra of XGFO supported this fact [32,33]. Scherer’s Equation (3), given below, is used to gain more information about crystallite size and interplanar distance [42]

(3) $$D=\frac{0.9\lambda }{\beta \cos\theta }$$

*D* here is the crystal’s size, *λ* stands for wavelength used (value is 1.54 A°), *β* indicates the half-width of the most intense peak, and *θ* represents diffraction angle. The Scherer crystallite size was found to be 22 nm for α-Fe_2_O_3_ NPs and 18.5 nm for XGFO. The contraction in size from 22 nm to 18.5 nm suggests that XGFO not only stabilized the α-Fe_2_O_3he_ NPs but also assisted in the reduction of Fe^3+^ ions. The interplanar distance d_311_ also increased from 2.50 in α-Fe_2_O_3_ to 2.52 in XGFO, which also supports the functionalization process.

**Figure 3:**
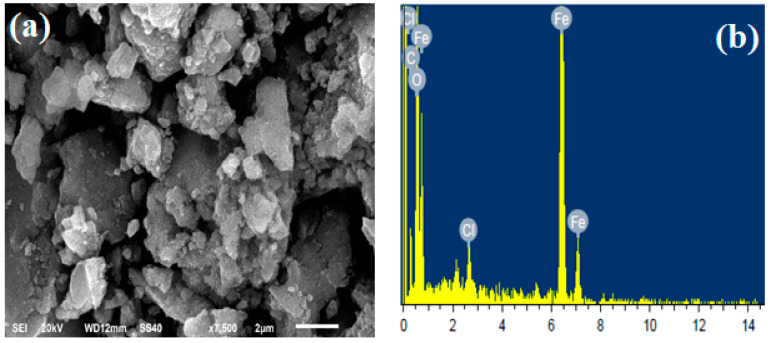

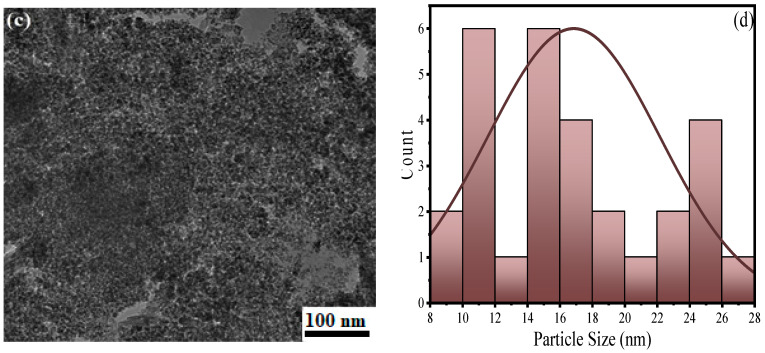
SEM images of (**a**) XGFO, (**b**) EDX spectra of XGFO, (**c**) TEM image of XGFO BNC, (**d**) Gaussian distribution of particle size.

Surface morphological changes in a material are observed through SEM during solid-state reactions. Figure 3a represents the SEM image of XGFO which possess an irregular distorted flaky morphology due to anchoring of an amorphous layer of biopolymer. The elemental composition of the material was confirmed by EDX analysis (Figure 3b), which shows the presence of C (24.62%), O (46.98%) and Fe (28.40%) distributed equally in the whole material. More insight into the particle size and morphology was observed by TEM analysis, which is given by Figure 3c. The results suggested the synthesized BNC material XGFO consists of small spherical particles with an ordered distribution. Figure 3d gives the Gaussian distribution of particle sizes and suggests the average particle size is around 17 nm.

**Figure 4:**
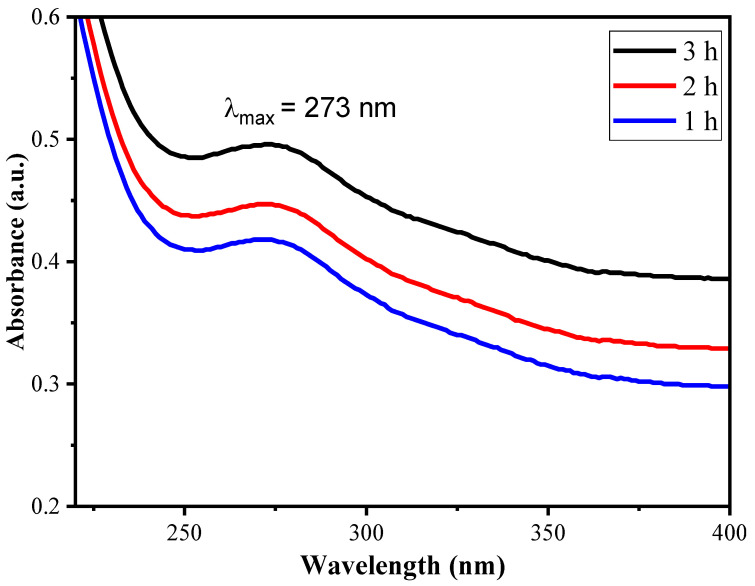
UV-Vis plot of XGFO in wavelength range of 200–400 nm.

UV-Vis spectroscopy with a wavelength range of 200–400 nm was used to get information about optical properties and growth in nanoparticle nucleation in a biopolymer matrix during synthesis. From Figure 4, it can be seen that with an increase in time, the absorbance value increases continuously, suggesting continuous growth of the α-Fe_2_O_3_ NPs with absorbance maxima at 273 nm. The transition belongs to the n–π* charge transfer from 2p orbital of –OH groups of XG to empty 3d orbital of Fe^2+^, resulting in the formation of α-Fe_2_O_3_ NPs [43].

**Figure 5:**
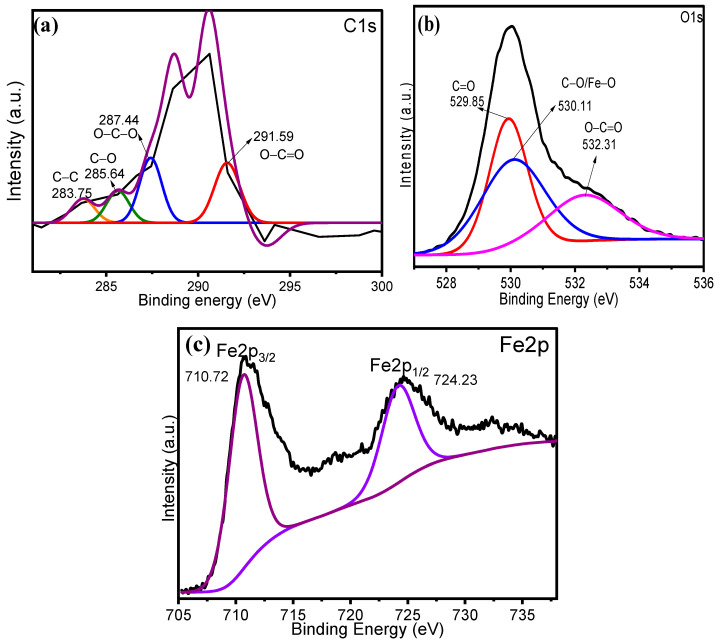
Deconvoluted XPS spectra of (**a**) C1s, (**b**) O1s and (**c**) Fe2p, corresponding to XGFO BNC.

To further evaluate the chemical oxidation states of individual elements in the XGFO BNC, XPS analysis was taken into consideration. Figure 5a represents the deconvoluted XPS spectra for C1s, which show four peaks at 283.75, 285.64, 287.44 and 291.59 eV, corresponding to C–C, C–O, O–C–O and O–C=O groups associated with the XG biopolymer [44]. The XPS spectra given in Figure 5b exhibit three deconvoluted peaks at 529.85, 530.11 and 532.31 eV, belonging to the C=O, C–O–Fe and O–C=O groups [38,44]. Thus, XG encapsulated the α-Fe_2_O_3_ NPs through C-O-Fe bonds in XGFO BNC. The XPS spectra for Fe2p shown in Figure 5c exhibit two peaks at 710.72 and 724.23 associated with the Fe2p_3/2_ and Fe2p_1/2_ chemical states, respectively [45]. The obtained XPS results are very well supported by the reported studies [46].

### 3.2. Optimization of Variables and Contour Plots

**Figure 6:**
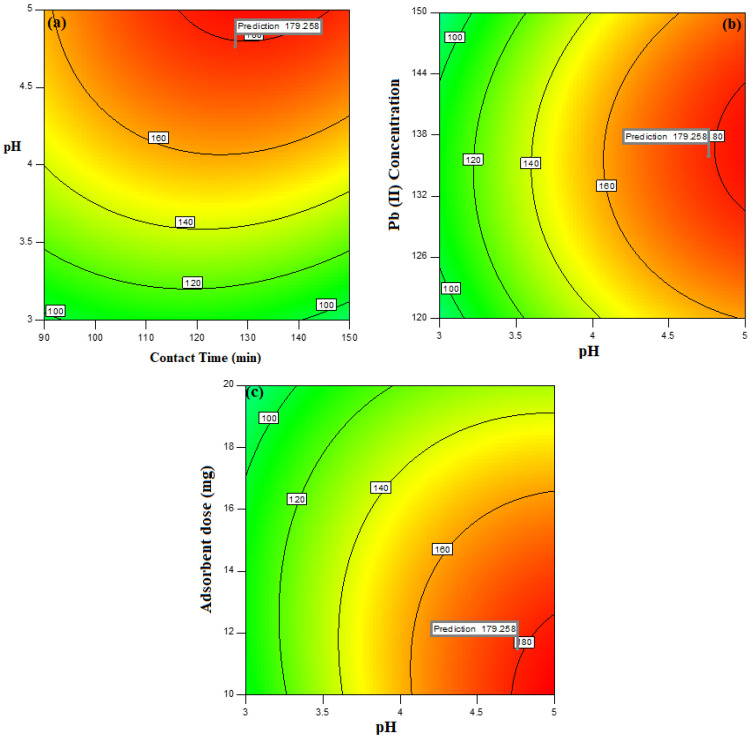
2D interactive contour plot depicting variation of adsorption capacity with respect to (**a**) contact time vs. pH, (**b**) pH vs. Pb (II) concentration and (**c**) adsorbent dose vs. pH.

The 2D interactive contour plots explaining the effect of two variables simultaneously were studied to evaluate the optimized values. Figure 6a represents the 2D contour plot between contact time between adsorbate and adsorbent and the pH of adsorbate solution with respect to adsorption capacity. It can be seen that with increase in contact time from 5 to 120 min, there is an increase in the adsorption capacity; beyond that, the capacity becomes constant, suggesting the attainment of equilibrium at 120 min. Similarly, it was observed that with an increase in pH from 1 to 4.8, the adsorption capacity also increases, suggesting an increase in rate of Pb (II) uptake up to pH 4.8; beyond these values, it starts decreasing [47]. Figure 6b represent the 2D graph for Pb (II) concentration vs. pH of the system on adsorption capacity of XGFO. It can be seen that as the Pb (II) concentration increases up to 135 mg L^−1^, the adsorption capacity of XGFO also increases and with a further increase in Pb (II) concentration, decreases. It was observed that an increase in Pb (II) concentration is reflected in the increase in adsorption capacity up to 135 mg L^−1^, and beyond this value adsorption capacity decreases, suggesting the occupation of all the reactive sites on the surface of adsorbent by Pb (II) ions. Figure 6c represents the adsorbent dose (mg) vs. pH of the system on the adsorption capacity of the material. It was observed that as the adsorbent dose increases from 5 mg to 11 mg, the adsorption capacity increases and decreases beyond these values due to the agglomeration of nanomaterial [48]. Therefore, 11 mg of adsorbent amount was chosen for further adsorption experiments. Based on the output of the experiments, it was concluded that 135 mg L^−1^ Pb (II) in 11 mg of XGFO at pH 5 for 120 min of contact time results in 179.26 mg g^−1^ adsorption capacity.

### 3.3. Adsorption Isotherm

Interactions between Pb (II) and adsorbent is acknowledged on the basis of adsorption isotherms. Adsorption data was applied to mathematical equations of Langmuir, Freundlich, Dubinin–Radushkevich (D–R) and Temkin models of isotherms at 303, 313, 323 and 333 K temperature. The mathematical equations of these models are given as [49,50,51,52]

(4) $$\frac{C_{e}}{q_{e}}=\frac{1}{{}_{\;}q_{m}K_{L}\;\;}+\frac{C_{e}\;}{q_{m}}$$

(5) $$\ln q_{e}=\frac{1}{{}_{\;}n\;\;}\ln C_{e}+\ln K_{F}$$

(6) $$\ln q_{e}=\ln q_{m}-K_{D-R}\varepsilon^{2}$$

(7) $$E=\frac{1}{\sqrt{2K_{D-R}}}$$

(8) $$\varepsilon =RT\left(1+\frac{1}{C_{e}}\right)$$

(9) $$q_{e}=B\ln A+B\ln C_{e}$$

Here, *q_m_* is the maximum monolayer adsorption capacity, *K_L_* is the Langmuir constant, *C_e_* denotes the Pb (II) at equilibrium, and *q_e_* the adsorption capacity of the material at equilibrium. The Langmuir model is applied when the adsorption data are simulated with a homogeneous surface consisting of a monolayer adsorption of Pb (II) on the surface of XGFO. The value of *n* is an integer measuring the favourability of the adsorption reaction, e.g., *n* greater than unity means adsorption is favourable, while *n* less than unity reflects lesser favourability of adsorption of Pb (II) towards XGFO. The Freundlich adsorption isotherm model deals with the concept of adsorption on a reversible heterogeneous surface. The D–R isotherm deals with the distribution of adsorption energy in a Gaussian way over a heterogeneous surface. *K_D–R_* represents the D–R constant (mol^2^ KJ^−2^), E means free energy per molecule, and ε indicates the D–R isotherm constant. The Temkin constant A belongs to the binding energy, while B belongs to the heat of the reaction. The Temkin isotherm model is applied to study adsorbent–adsorbate interactions. Two assumptions are made under this isotherm: (i) the heat of adsorption decreases linearly, not logarithmically (ii) distribution of binding energies is uniform.

**Figure 7:**
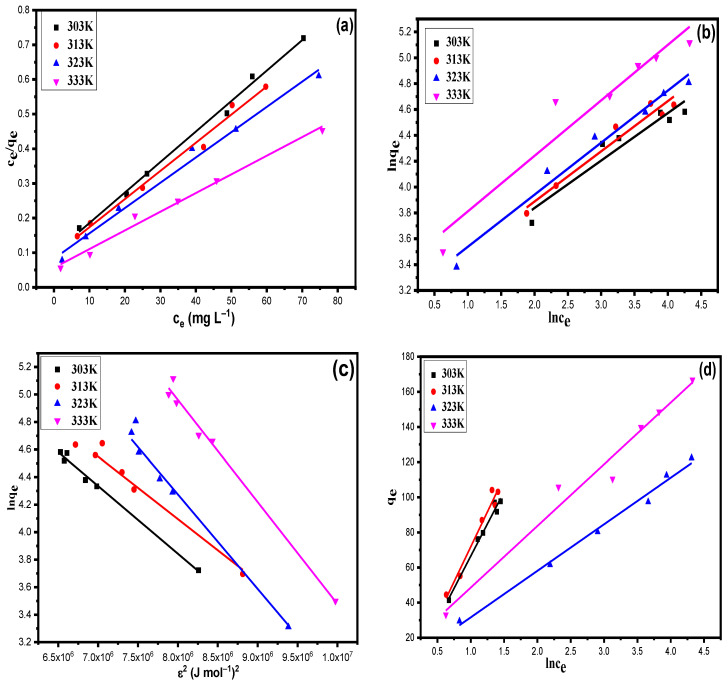
(**a**) Langmuir, (**b**) Freundlich, (**c**) D–R and (**d**) Temkin adsorption isotherms for Pb (II) on XGFO at 303 K, 313 K, 323 K and 333 K (adsorbent dose = 11 mg and pH 4.8).

The obtained data after adsorption reactions performed at optimized reaction conditions were applied to Equations (4)–(9) using the linear regression method, and the obtained results are given Figure 7a–d and Table 1. From the results given in Table 1, it was observed that with the lowest value of RMSE (0.018 at 303 K, 0.015 at 313 K, 0.013 at 323 K and 0.010 at 333 K) and the highest value of regression coefficient R^2^ (0.99 for all temperature ranges), the Langmuir model was found to be the best isotherm that simulates the adsorption data with minimum error. The maximum monolayer adsorption capacity was found to be 117.45 mg g^−1^ at 303 K, 126.23 mg g^−1^ at 313 K, 145.12 mg g^−1^ at 323 K and 191.27 mg g^−1^ at 323 K. The value of the Langmuir constant KL was found to be 0.074 L mg^−1^ at 303 K, 0.085 L mg^−1^ at 313 K, 0.089 L mg^−1^ at 323 K and 0.095 L mg^−1^ at 333 K. The increase in *K_L_* values with respect to temperature suggests the high affinity of XGFO towards Pb (II) and, as a result, a high adsorption capacity obtained at high temperature. The value of *n* obtained by the Freundlich model was found to be greater than 1 at all temperature ranges, suggesting the favourability of the adsorption reaction between Pb (II) and XGFO. The increase in value of *n* with temperature suggests the high favourability of Pb (II) uptake by XFGO at high temperature, reflecting a high adsorption capacity. The value of mean free energy per molecule (*E*) calculated by the D–R model suggests the adsorption reaction of Pb (II) to XGFO as chemisorption, i.e., the adsorbent removes the Pb (II) from wastewater by formation of chemical bonds and thus avoids secondary pollution.

### 3.4. Adsorption Kinetics

In order to evaluate the rate-determining step, the kinetic data was applied to various kinetic models such as the Lagergren pseudo-first-order, pseudo-second-order, Elovich and Weber-Morris Intra-particle diffusion. The linear mathematical expressions of these models are given by Equations (10)–(13) [53,54,55,56]:

(10) $$\log\left(q_{e}-q_{t}\right)=\log q_{e}-\frac{k_{1}}{2.303}t$$

(11) $$\frac{t}{q_{t}}=\frac{1}{k_{2}q_{e}^{2}}+\frac{t}{q_{e}}$$

(12) $$q_{t}=\frac{1}{\beta }\ln t+\frac{1}{\beta }\ln\left(\alpha \beta \right)$$

(13) $$q_{t}=k_{\mathrm{int}}t_{}^{0.5}+C$$

**Figure 8:**
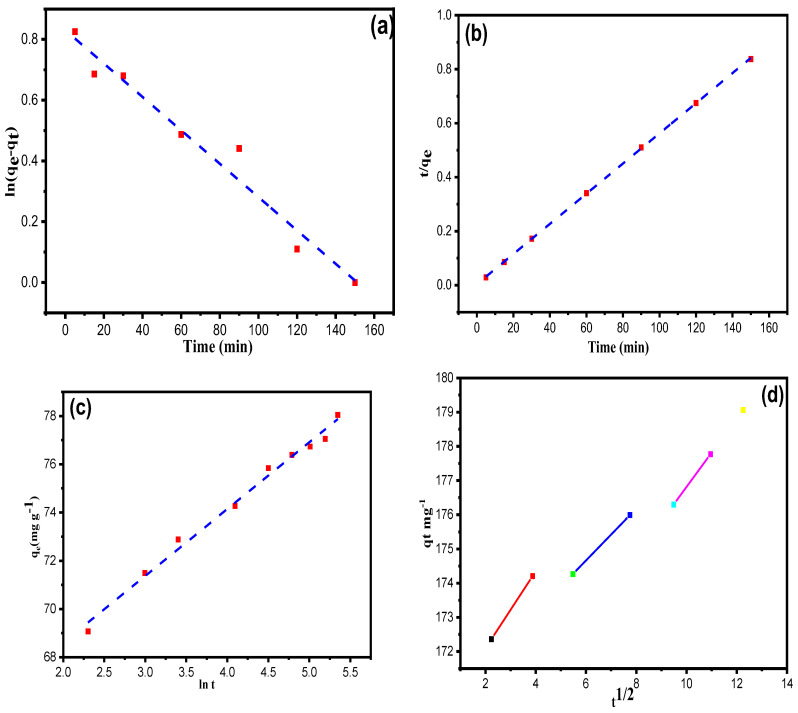
(**a**) Pseudo-first-order, (**b**) Pseudo-second-order, (**c**) Elovich and (**d**) Intraparticle Diffusion model for the removal of Pb (II) onto XGFO BNC with optimum concentration (135 mg L^−1^) and pH at 323K.

Here, *q_e_* and *q_t_* (mg g^−1^) specify the adsorption capacity of XGFO at equilibrium and at time *t*, and *k*_1_ represents pseudo-first-order rate constant (min^−1^). *k*_2_ indicates pseudo-second-order rate constant (g mg^−1^ min^−1^). *α* is the initial degradation rate (mg g^−1^ min^−1^); *β* indicates the degradation constant (g mg^−1^), and *k*_int_ represents the intraparticle diffusion rate constant (mg g^−1^ min^1/2^). The results obtained after the simulation of kinetic data with Equations (10)–(13) are listed in Table 2 and Figure 8a–d. It can be inferred that with a high value of the regression coefficient (R^2^ = 0.99) and low value of RMSE (0.019), the pseudo-second-order model defines well the uptake process of Pb (II) by XGFO BNC through chemical adsorption. The polyfunctional –OH groups on the surface of the material efficiently bind the Pb (II) ions through ligand-to-metal charge transfer LMCT and thus form a chemical bond. The obtained experimental value of adsorption capacity (*q_e_*, exp) for the pseudo-second-order has been found in close agreement with the calculated values as compared with other models. The data in Table 2 for the Elovich model represent an analogue of the results with the correlation coefficients whose value obtained is low in comparison to the pseudo-first-order and pseudo-second-order, which accounts for the inadmissible factor of this model for the degradation system. Multi-linearity was observed in the intraparticle diffusion model, suggesting the involvement of two or more steps in the uptake of Pb (II) by XGFO. The first linearity indicates the instantaneous uptake of Pb (II), and the second linearity indicates the uptake of Pb (II) at a controlled rate. Finally, at the equilibrium stage, the low concentration of Pb (II) poses a slowdown in the intraparticle diffusion rate.

### 3.5. Thermodynamic Aspect of Adsorption Reaction

The thermodynamic aspect of the adsorption reaction was investigated to observe the nature of the reaction with respect to heat. In this regard, Equations (14)–(16) were applied to the data obtained after performing adsorption reaction with respect to temperature and results obtained are listed in Table 3.

(14) $$K_{c}=\frac{C_{ad}}{C_{e}}$$

(15) $$\ln K_{c}=-\frac{\Delta H_{}^{0}}{R}+\frac{\Delta S_{}^{0}}{RT}$$

(16) $$\Delta G_{}^{0}=\Delta H_{}^{0}-T\Delta S_{}^{0}$$

**Figure 9:**
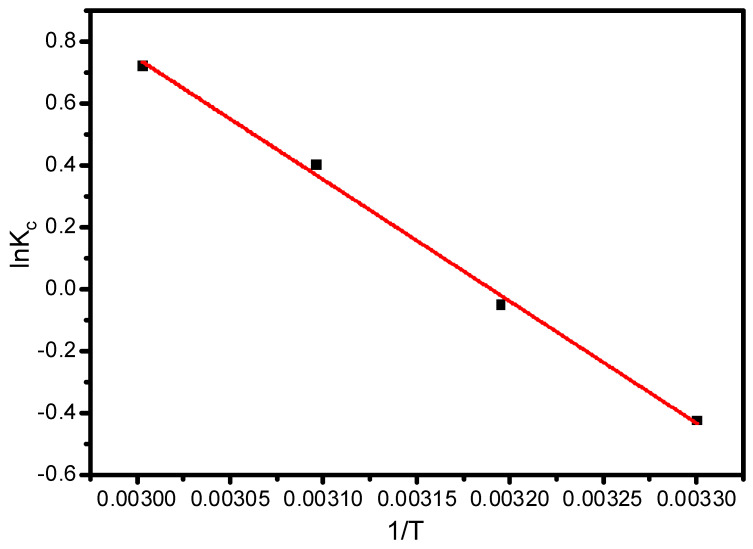
Thermodynamic plot for ln Kc vs. 1/T for the estimation of enthalpy and entropy of adsorption reaction of Pb (II) and XGFO BNC.

*K_c_* stands for the distribution coefficient obtained by Equation (14) at various temperatures, and *C_e_* and *C_ad_* represent concentrations at liquid and solid phases (mg L^−1^), respectively. The obtained thermodynamic plot between ln *K_c_* vs. 1/T has been given in Figure 9. The slope and intercept of Figure 9 computed the enthalpy and entropy of the reaction, which are given as Δ*H*^0^ = 29.72 KJ mol^−1^ and Δ*S*^0^ = 0.112 KJ mol^−1^ K^−1^. The Gibbs free energy Δ*G*^0^ was calculated using Equation (16), and is given as −4.22 KJ mol^−1^ at 303 K, −5.34 KJ mol^−1^ at 313 K, −6.45 KJ mol^−1^ at 323 K, and −7.57 KJ mol^−1^ at 333 K, respectively. The negative values of Δ*G*^0^ indicate that the interaction of Pb (II) and XGFO is spontaneous and feasible. The feasibility of the reaction increases with increase in temperature.

### 3.6. Adsorption Mechanism

**Scheme 2:**
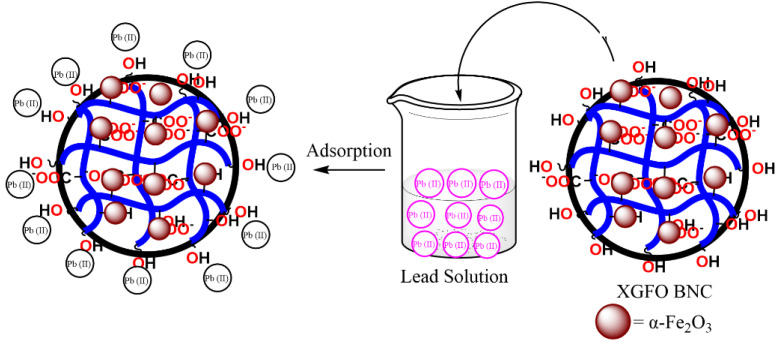
Illustration of Pb (II) uptake mechanism by XGFO BNC under optimized condition.

**Figure 10:**
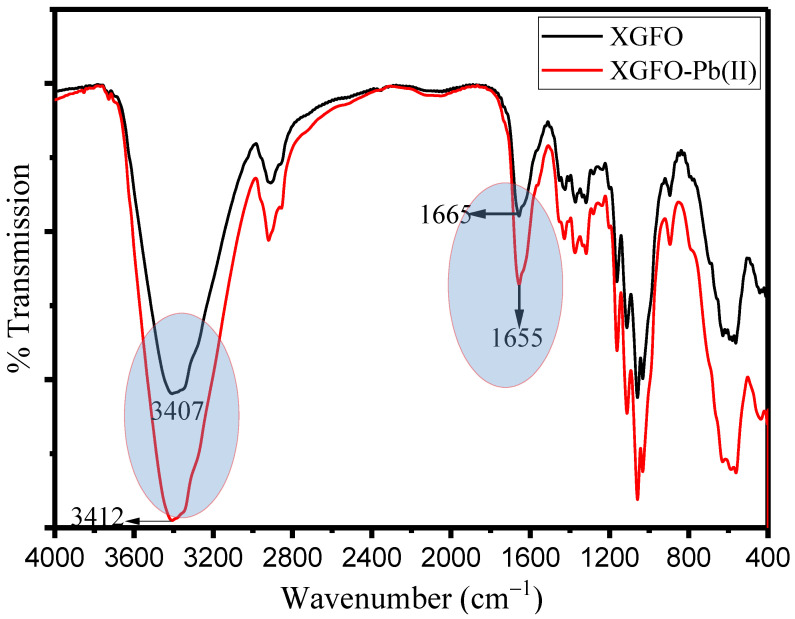
FTIR spectra of XGFO before and after adsorption of Pb (II).

Scheme 2 represents the adsorption mechanism of Pb (II) on synthesized XGFO BNC. Figure 10 represents the FTIR spectra of pristine XGFO and Pb (II)-loaded XGFO. It can be seen that after Pb (II) adsorption the frequency of the carbonyl group (C=O) shifted from 1665 to 1655 cm^−1^; similarly, for hydroxyl groups (–OH), it shifted from 3407 cm-1 to 3412 cm^−1^, which suggests that both –COOH and –OH groups are involved in Pb (II) by XGFO. Based on the FTIR analysis, there may be two ways by which Pb (II) can be adsorbed on XGFO: (i) through chemical bond formation, and (ii) through electrostatic attractions. At pH ≅ 5, the negatively charged surface of XGFO with deprotonated hydroxyl and carboxy groups tends to attract Pb (II) ions through electrostatic interactions, or H^+^ ions on –OH and –COOH groups can be exchanged with Pb (II) ions, leading to the formation of the complex with O atom of the adsorbent. The energy from D–R model is calculated as 11.12 KJmol^−1^ at 298 K, 12.79 KJmol^−1^ at 308 K, 13.04 KJmol^−1^ at 318 K, and 14.22 KJmol^−1^ at 328 K, which is greater than 8 KJmol^−1^, suggesting that the adsorption of Pb (II) by XGFO is chemisorption. It was therefore concluded that Pb (II) sequestration from wastewater by XGFO is purely through the formation of chemical bonds, which is not susceptible to causing secondary pollution. Similar adsorption mechanisms were also proposed by magnetic Co_0.6_Fe_2.4_O_4_ micro-particles [57].

### 3.7. Desorption and Regeneration

**Figure 11:**
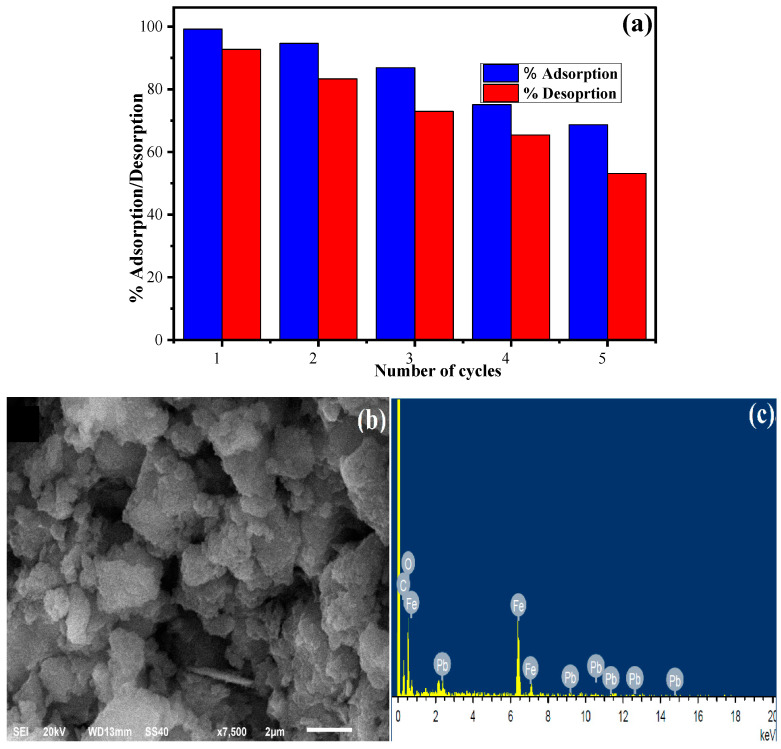
(**a**) Adsorption-desorption bar graph for regeneration of XGFO toward Pb (II) adsorption, (**b**,**c**) SEM-EDX spectra of Pb (II)-loaded XGFO at optimized conditions.

Desorption and regeneration of the adsorbent is an important process in view of economical and practical applications. The adsorption experiments were performed by taking 135 mg L^−1^ Pb (II) in 11 mg of XGFO at pH 5 for 120 min of contact time. After the completion of the reaction, the material was collected through centrifugation, dried and again immersed in a 20 mL solution of 0.2 M HCl solution. The solution was stirred for 120 min, and then the material was collected, washed with distilled water and dried in a hot air oven at 60 °C. The supernatant collected was analyzed for the concentration of Pb (II) desorbed using AAS. This process was repeated till five consecutive cycles of adsorption-desorption, and the results obtained are given in Figure 11a. The regeneration efficiency *R* (%) of the synthesized adsorbent material can be calculated using Equation (17):

(17) $$R\;(\%)=\left(\frac{C_{0}-C_{e}}{C_{0}}\right)\times 100$$

where *C*_0_ and *C_e_* (mg L^−1^) are the initial and final concentrations of Pb (II), respectively. In the first cycle the adsorption-desorption was calculated as 99.23% and 92.75%, which reduced to 68.67% adsorption and 53.15% desorption in the fifth cycle. The results from adsorption–desorption experiments suggest that the synthesized material can be effectively recovered and reused for the removal of Pb (II) from wastewater for up to five consecutive cycles. Figure 11b,c represent the SEM-EDX of Pb (II)-loaded XGFO after five cycles of adsorption-desorption, which indicates an irregular flaky morphology. EDX spectra confirm the loading of Pb (II) on the XGFO with atomic percentage as C (43.28%), O (43.64%), Fe (12.62%) and Pb (0.45%), respectively.

### 3.8. Comparison with Literature

The present study was compared with the data reported in literature and the results are given in Table 4. It can be seen that the present study showed a higher adsorption capacity towards Pb (II) as compared to other adsorbent material with respect to contact time and adsorbent dose.

## 4. Conclusions

The present study demonstrated the synthesis of a green functional nanocomposite material based on α-Fe_2_O_3_ immobilized with xanthan gum biopolymer. The FTIR analysis suggested the functionalization and stabilization of α-Fe_2_O_3_ through formation of Fe^2+^–OH-type coordinated bonds. The XRD analysis represented a trigonal rhombohedral structure in which Fe^3+^ is bonded to six equivalent O^2−^ atoms to form a mixture of distorted face, edge, and corner-sharing FeO_6_ octahedra. The Scherer formula suggested the crystallite size as 22 nm for α-Fe_2_O_3_ NPs and 18.5 nm for XGFO. The morphological studies suggested the formation of porous flakes due to presence of a biopolymer of elemental composition. The TEM analysis was also found to be in good agreement with the Scherer formula and calculated the particle size as 17 nm. The applicability of the material was explored as an adsorbent for the removal of toxic Pb (II) from wastewater followed by optimization of various reaction variables. The optimized values of reaction variables were found to be a contact time of 120 min, pH of 5, Pb (II) concentration of 135 mg L^−1^ and adsorbent dose of 15 mg. The adsorption reaction data were best simulated with the Langmuir isotherm model and the value of the maximum monolayer adsorption capacity was found to be 117.45 mg g^−1^ at 303 K, 126.23 mg g^−1^ at 313 K, 145.12 mg g^−1^ at 323 K and 191.27 mg g^−1^ at 323 K, with the highest value of correlation coefficient (R^2^ = 0.99). The sequestration reaction of Pb (II) was best explained by the pseudo-second-order model, suggesting the uptake of Pb (II) by XGFO was via chemisorption reaction, which suggests that the material is remaining Pb (II) without creating secondary pollution. The thermodynamic aspect of the adsorption reaction suggests that the reaction is endothermic and feasible. The feasibility of the reaction increases as the temperature of the reaction increases. The regeneration studies suggested that the material is highly effective towards Pb (II) removal and can be effectively reused by using 0.2 M HCl solution.

## Figures and Tables

**Table 1:** Adsorption isotherm data obtained through the linear regression method for removal of Pb (II) onto XGFO BNC.

Model	Parameters	303 K	313 K	323 K	333 K
Langmuir	Qm (mg g^−1^)	117.45	126.23	145.12	191.27
	*K_L_* (L mg^−1^)	0.074	0.085	0.089	0.095
	R^2^	0.99	0.99	0.99	0.99
	RMSE	0.018	0.015	0.013	0.010
Freundlich	*K_F_* (mg g^−1^)	24.13	24.42	24.87	28.67
	*n*	2.05	2.37	2.58	2.84
	R^2^	0.85	0.94	0.96	0.92
	RMSE	0.062	0.057	0.048	0.069
D–R	*q_m_* (mg g^−1^)	232.45	235.78	251.73	273.69
	*K_D–R_* (mol^2^ J^−2^)	3.78 × 10^−7^	4.69 × 10^−7^	5.54 × 10^−7^	6.86 × 10^−7^
	*E* (J mol^−1^)	1112.19	1279.47	1304.35	1422.87
	R^2^	0.96	0.82	0.97	0.91
	RMSE	0.21	0.92	0.72	0.79
Temkin	*A* (L mg^−1^)	0.624	0.859	1.342	1.521
	*B* (J mol^−1^)	105.45	98.49	107.23	92.85
	R^2^	0.91	0.97	0.99	0.97
	RMSE	2.13	1.75	1.89	1.05

**Table 2:** Kinetic data obtained through linear regression for removal of Pb (II) by XGFO BNC at 323 K temperature.

Parameters	Kinetic Models			
	Pseudo-First-Order	Pseudo-Second-Order	Elovich	Intraparticle Diffusion
*q_e_* (mg g^−1^)	6.76	166.66	0.602	6.579
*k*_1_/*k*_2_/*k*_p_/*k*_int_	0.011	111.102	171.23	0.566
R^2^	0.95	0.99	0.955	0.88
RMSE	0.039	0.019	0.053	0.25

**Table 3:** Thermodynamic parameters for the adsorption of Pb (II) on XGFO at 303 K, 313 K, 323 K and 333 K.

Thermodynamics Study	∆*H* (KJ mol^−1^)	∆*S* (KJ mol K^−1^)	∆*G* (KJ mol^−1^)			
			303 K	313 K	323 K	333 K
	29.72	0.112	−4.22	−5.34	−6.45	−7.57

**Table 4:** Comparison with other materials in the literature.

S.N.	Adsorbent Material	Adsorption Capacity (mg g^−1^)	Optimum Time (min)	Adsorbent Dose (mg)	References
1	Fe_3_O_4_@BDC@AGPA	157	180	20	[58]
2	NaxFeyMgiTiOz	20.3	400	10	[59]
3	Mg–Al LDH/rGO	116.2	180	100	[60]
4	TA-KCC-1/Chi-OLA NC	168	100	20	[61]
5	P(AAm-co-IA)/MWCNTs	101.01	90	50	[62]
6.	Cs-SB@SrFe_12_O_19_	132	120	20	[63]
7	Cellulose sulfate/chitosan	137.8	40	100	[64]
8	CS-ag-CM	182.5	40	50	[47]
9	XGFO	191.27	120	15	Present Study

## Data Availability

Data presented in this study are available on request from the corresponding author.
